# Dysbiosis and Restoration Dynamics of the Gut Microbiome Following Therapeutic Exposure to Florfenicol in Snubnose Pompano (*Trachinotus blochii*) to Aid in Sustainable Aquaculture Production Strategies

**DOI:** 10.3389/fmicb.2022.881275

**Published:** 2022-05-30

**Authors:** T. G. Sumithra, Krupesha S. R. Sharma, Suja Gangadharan, Gayathri Suresh, Vishnu Prasad, P. V. Amala, P. Sayooj, Ambarish P. Gop, M. K. Anil, Prasanna Kumar Patil, Gopalakrishnan Achamveetil

**Affiliations:** ^1^Marine Biotechnology Division, Indian Council of Agricultural Research (ICAR)-Central Marine Fisheries Research Institute, Kochi, India; ^2^Vizhinjam Regional Centre of ICAR-Central Marine Fisheries Research Institute, Thiruvananthapuram, India; ^3^Aquatic Animal Health and Environment Division, ICAR-Central Institute of Brackishwater Aquaculture, Chennai, India

**Keywords:** fish health, environment, antimicrobial resistance, kanamycin, antibiotics, postwithdrawal

## Abstract

Information on unintended effects of therapeutic exposure of antibiotics on the fish gut microbiome is a vital prerequisite for ensuring fish and environmental health during sustainable aquaculture production strategies. The present study forms the first report on the impact of florfenicol (FFC), a recommended antibiotic for aquaculture, on the gut microbiome of snubnose pompano (*Trachinotus blochii*), a high-value marine aquaculture candidate. Both culture-dependent and independent techniques were applied to identify the possible dysbiosis and restoration dynamics, pointing out the probable risks to the host and environment health. The results revealed the critical transient dysbiotic events in the taxonomic and functional metagenomic profiles and significant reductions in the bacterial load and diversity measures. More importantly, there was a complete restoration of gut microbiome density, diversity, functional metagenomic profiles, and taxonomic composition (up to class level) within 10–15 days of antibiotic withdrawal, establishing the required period for applying proper management measures to ensure animal and environment health, following FFC treatment. The observed transient increase in the relative abundance of opportunistic pathogens suggested the need to apply proper stress management measures and probiotics during the period. Simultaneously, the results demonstrated the inhibitory potential of FFC against marine pathogens (vibrios) and ampicillin-resistant microbes. The study pointed out the possible microbial signatures of stress in fish and possible probiotic microbes (*Serratia* sp., *Methanobrevibacter* sp., *Acinetobacter* sp., and *Bacillus* sp.) that can be explored to design fish health improvisation strategies. Strikingly, the therapeutic exposure of FFC neither caused any irreversible increase in antibiotic resistance nor promoted the FFC resistant microbes in the gut. The significant transient increase in the numbers of kanamycin-resistant bacteria and abundance of two multidrug resistance encoding genes (K03327 and K03585) in the treated fish gut during the initial 10 days post-withdrawal suggested the need for implementing proper aquaculture effluent processing measures during the period, thus, helps to reduce the spillover of antibiotic-resistant microbes from the gut of the treated fish to the environment. In brief, the paper generates interesting and first-hand insights on the implications of FFC treatment in the gut microbiome of a marine aquaculture candidate targeting its safe and efficient application in unavoidable circumstances. Implementation of mitigation strategies against the identified risks during the initial 15 days of withdrawal period is warranted to ensure cleaner and sustainable aquaculture production from aquatic animal and ecosystem health perspectives.

## Introduction

Aquaculture is the fastest-growing food-producing sector globally and makes a significant contribution to poverty alleviation, food security, and income generation across the globe (Walker and Winton, 2010). Since most of the development in the aquaculture sector has occurred during the last 50 years, the sustainability of aquaculture practices, both in terms of economics and environmental health, has evolved into a growing concern (Boyd et al., 2020). The increasing reliance on farmed fish for human nutrition, as well as the persistent and growing challenge of infectious diseases often leads to a heavy reliance on the use of antibiotics for prophylactic or therapeutic measures in the aquaculture industry (Schmidt et al., 2017). Excessive or indiscriminate antibiotics use forms a significant constraint in achieving sustainable aquaculture production. Antibiotic exposure can cause several dysbiotic events in the gut microbiota of the host, with a considerable influence on host immunity, development, nutrition, and health (Pennycook and Scanlan, 2021). Studies in terrestrial animals, including humans, have demonstrated the wide-ranging implications of antibiotic therapy on the ecology and evolution of the gut microbiota, with pronounced effects on host’s health and welfare (Pennycook and Scanlan, 2021). The current research interest on gut dysbiotic events focuses mainly on mammalian models, while studies on non-mammalian models such as the fish are scarce. Furthermore, the role of the fish microbiome in host’s health is less established to date (Schmidt et al., 2017). The effects of antibiotic administration on the fish gut microbiome and the possible outcomes are still under-study (Kokou et al., 2020). The corresponding information on major antimicrobials in tropical marine fish species is completely lacking. The information on the impacts of antibiotics on the gastrointestinal microbiota of aquaculture candidate fish species can be directly applied to exploring sustainable aquaculture production strategies (Navarrete et al., 2008).

The application of antibiotics may cause the emergence of antibiotic resistance in gut microbes of the host, raising concerns about consumer and environmental safety through the contamination by aquaculture wastes carrying antibiotic-resistant bacteria (Cabello et al., 2013). Studies have demonstrated that the dysbioses in the gut microbiome in response to antibiotic treatment led to the emergence of antibiotic resistance in the gut (Shoemaker et al., 2001; Francino, 2016). Due to the vast density of bacterial cells and species richness, the gut microbiota is likely to be prone to horizontal gene transfer leading to the spread of antimicrobial-resistant genes between bacterial taxa in addition to their spread to incoming pathogenic microorganisms (Whittle et al., 2002; Sommer et al., 2009). In brief, the research on antibiotic effects on the gut microbiome is critical from agricultural and ecological perspectives and needs to be soon addressed to achieve sustainable aquaculture practices (Kokou et al., 2020).

The snubnose pompano (*Trachinotus blochii*) is one of the promising candidates among the different high-value marine tropical finfishes with fast growth rates and high market demand, therefore, are recommended for sustainable marine aquaculture practices (FAO et al., 2021). Incidences of infectious diseases, especially vibriosis, hamper many successful farming practices of pompano (Liu et al., 2004; Yu et al., 2018), forcing the farmers to rely on the use of different antimicrobials to treat or prevent the diseases. There are only four recommended antimicrobial compounds for aquaculture purposes, *viz*. sulfamerazine, oxytetracycline, sulfadimethoxine-ormetoprim, and florfenicol (FFC) (U.S. Food and Drug Administration [USFDA], 2008). Of these, FFC is the preferred antimicrobial since it has never been used in human medicine (Committee for veterinary medicinal products, 2001). The clinical efficacy of FFC through oral administration has been ratified in different farmed marine fish species against fish pathogens (Feng et al., 2018; San Martín et al., 2019). Similarly, while limited information is available on the effects of FFC treatment on the fish gut microbiome (Abdelhamed et al., 2019), no corresponding information is available on the marine fish gut microbiome. Given the above facts, the present study was envisaged to ascertain the impact of the therapeutic exposure of FFC on the gut microbiome of *T. blochii* through a combination of culture-dependent and culture-independent approaches with the following objectives: (1) to evaluate the influence of FFC exposure in *T. blochii* on the gut microbiota through both culture-dependent and independent techniques; (2) to understand the restoration dynamics of the gut microbiome following FFC treatment by evaluating the changes in cultivable bacterial density, diversity measures of *16S rRNA* amplicon-based metagenomics, and taxonomic microbial composition; (3) to outline the emergence of antimicrobial resistance in the gut microbes during FFC therapeutic interventions; and (4) to identify the prospective gut microbial biomarkers of FFC treatment in *T. blochii*.

## Materials and Methods

### Experimental Fish

Healthy snubnose pompano juveniles, with an average weight of 12 ± 0.62 g, were used in the present study. Fish (12 ± 0.62 g) were brought from the marine aquaculture facility of the Vizhinjam Regional Centre of ICAR-Central Marine Fisheries Research Institute (ICAR-CMFRI) and acclimatized for seven days in oval-shaped fibre reinforced plastic (FRP) tanks containing 700 L of de-chlorinated and continuously aerated water (temperature: 29.8 ± 0.54°C; pH: 7.5 ± 0.7; salinity: 18 ± 1.4). Fish were fed with floating pellet feed (Nutrila from Growel) at 5% biomass during acclimatization. The animals were allowed to acclimatize for seven days. Active feeding was observed after 2 days of stocking. Water quality parameters (ammonia, nitrite, and nitrate) were maintained to the optimal levels through 20% daily water exchange and siphoning out of the waste materials throughout the experimental period.

All the experiments involving live fish were done adhering to animal research reporting of *in vivo* experiments (ARRIVE) guidelines (du Sert et al., 2020), the guidelines of EU Directive 2010/63/EU for animal experiments (2019), and the U.K. Animals Scientific Procedures Act (1986). “The animal study was reviewed and approved by the ICAR-CMFRI, Kochi, India (CIBA/AINP-FH/2020-21).

### Preparation of Medicated Feed

The FFC medicated feed was prepared by surface coating the drug (Amit et al., 2017) onto a commercial.8 mm pellet feed (Nutrila from Growel). The proximate composition of the feed on a dry matter basis was 52% protein, 12% fat, and 1.5% fiber. The required dose of FFC (10 mg/kg biomass/day) was achieved by mixing 53 mg of FFC powder (Tokyo Chemical Industry, Japan), with 500 μL of fish oil, and then, coating uniformly onto 100 g of commercial feed. The mixture was kept for 5 min until the FFC oil mixture was evenly distributed on the pellets. The prepared feed was then dried at 300°C for 1 h. The control/non-medicated feed was processed in the same manner with surface coating only with fish oil.

### Experimental Design

For the experiments, the animals were randomly divided into two groups, *viz*. treatment and control groups. Each group was maintained in triplicate FRP tanks (25 fish per tank) containing 700 L of filtered water (salinity: 18%; temperature: 29°C) with continuous aeration. In the treatment group, fish were fed with FFC medicated feed at 10 mg/kg biomass for 10 days. After 10 days of medicated feeding, the fish in the treatment group were fed with non-medicated feed. Fish in the control group were fed with non-medicated feed throughout the experimental period. All the fish were fed at 2% of body mass per day, and it was confirmed that the fish had consumed all the feed. Fish were monitored daily for 30 days after the commencement of experiments. When fishes were removed from a tank for sampling, the quantity of feed administered to the tank was adjusted proportionally to maintain the therapeutic dose at 10 mg/kg of body weight (Soto et al., 2010).

### Sampling

Four fish/time points/tanks were randomly sampled at 5-day intervals from the commencement of the experiment. The external surface of the fish was cleaned using 70% ethanol to avoid surface microbial contamination. After opening the ventral surface, the entire gut was aseptically removed using clamps to prevent the release of intestinal contents. Gut samples of two fish from each tank were pooled and used for culture-dependent microbiological analysis. Gut samples from the remaining two fish in each tank were pooled and used for metagenomics analysis.

### Culture-Dependent Microbiological Analysis

The gut, along with the intestinal contents of each pool, was resuspended as 1 g/mL in sterile phosphate-buffered saline (PBS) and homogenized. Serial 10-fold dilutions of each homogenate were prepared and spread on Tryptic Soy Agar (TSA) and thiosulphate citrate bile salt sucrose agar (TCBS) plates supplemented with 1% sodium chloride (Himedia, India) in duplicates and incubated at 30°C for 48 h under aerobic conditions. The total viable count was expressed as the number of colony-forming units (CFU) per gram (Hovda et al., 2007). The viable counts of presumptive vibrios (mesophilic Vibrionaceae and other closely related vibrios) were enumerated after 48 h of incubation on TCBS agar (Bolinches et al., 1988). Further, each homogenate was added to six other selective culture plates containing either one of the six antibiotics, namely ampicillin, oxytetracycline, kanamycin, FFC, enrofloxacin, and meropenem (Guardabassi et al., 2002). The final concentration was 50 μg/mL for each antibiotic and 30 μg/mL for FFC (Kenzaka et al., 2006).

### Genomic DNA Isolation

For isolating the total microbiome DNA, the Qiamp stool kit (Qiagen) was utilized with certain modifications for reducing host DNA contamination (Wanka et al., 2018; Bruggeling et al., 2021). Initially, the gut, along with the intestinal contents of each pool, was homogenized in PBS (pH 7.4). The homogenate was then centrifuged at 1,000 rpm for 10 min. The supernatant was taken as a source of loosely associated bacteria with the target tissues. The bacteria strongly associated with the tissues were separated using a detergent solution (0.9% saline with 1% (w/v) Triton X 100) and collected with a pipette (Bruggeling *et al.*, 2021). These solutions (representing loosely and strongly associated bacteria of the target tissues) were mixed and centrifuged at 12,000 rpm for 10 min. The pellet representing loosely and strongly associated bacteria (Wanka et al., 2018; Bruggeling et al., 2021) was further processed for DNA isolation using DNeasy Blood and Tissue Kit (Qiagen) following the manufacturer’s protocol. The concentrations of DNA were measured using the Qubit Fluorimeter (V.3.0) and preserved at –20°C until needed.

### Amplification and Next-Generation Sequencing

The hypervariable V3–V4 region of the prokaryotic 16SrRNA gene from the total bacterial DNA (10 ng) was amplified using the primers, *viz.* Pro341F (5′-CCTACGGGNBGCASCAG-3′) and Pro805R (5′-GACTACNVGGGTATCTAATCC-3′) (Takahashi et al., 2014). The amplified product was gel-purified to remove non-specific amplification, if there is any. Metagenomic library preparation was done using the NEBNext Ultra DNA library preparation kit (New England Biolabs) using equimolar quantities of PCR amplicon (5 ng). The library quantity and quality were estimated in Agilent 2200 TapeStation. The sequencing was then performed on an Illumina HiSeq 2500 platform (2 × 300 paired-end sequencings) (AgriGenome Labs Private Limited, Kochi, India). The high-quality samples were refined and used for metagenomics analysis.

### Metagenomics Analysis

The raw reads generated were demultiplexed and evaluated for quality using the FastQC tool (version 0.11.8) with default parameters. The base quality (Phred Score; Q), adapter dimers, GC content, base composition, and ambiguous bases (apart from A, T, G, and C) were thoroughly scrutinized. The forward and reverse primer sequences were maintained to get all the possible *16S rRNA* gene sequence information. Further downstream analysis was done using the Quantitative Insights into Microbial Ecology pipeline (QIIME2™ version 2021.4.0) (Bolyen et al., 2019). Demultiplexed pair-end reads were merged, filtered, and denoised using the Divisive Amplicon Denoising Algorithm 2 (DADA2) (Callahan et al., 2016). An alpha rarefaction curve was then generated to ensure that the relation between the read depth and new taxon detection approached an asymptote in all the samples. The Naive Bayesian classifier against the SILVA database version 138 was then applied to assign the taxonomic information on the obtained amplicon sequence variants (ASVs), in which operational taxonomic units (OTUs) were clustered by 99% homology. The ANCOM plugin was used to calculate the relative abundance of each taxonomic level within the samples. The diversity measures were estimated in QIIME2 using the core metrics pipeline. The PICRUSt2 tool was explored to predict metagenome functions, *viz*. KEGG orthologs and pathways (Douglas et al., 2020).

### Statistical Analysis

The normality and homogeneity of variance of different data sets were initially checked using the Shapiro–Wilk test and the Levene test, respectively. One-way ANOVA followed by Tukey’s HSD test was used to compare the viable counts of bacteria in the various culture media between different days of antibiotic exposure with *P* values of <0.05 and <0.01 set to represent significant and highly significant differences, respectively. The OTU abundance of the taxa accounting for >0.01% was used to create the relative abundance plot. The α-diversity measures in terms of OTU richness, evenness, abundance-based coverage estimator (ACE), chao1, Shannon index, and Simpson index were then calculated using the Past software (version 3.5.2) (Hammer et al., 2001). Differences in α-diversity indices in microbial diversity on different days of antibiotic exposures were determined with an ANOVA/Krusswallis test based on the normality of the data. The similarity index of different days of antibiotic exposures was calculated using the Bray–Curtis distance method and compared through PERMANOVA analysis in PAST 3.5.2 software. The average Bray–Curtis similarity index of each group was analyzed through the hierarchical clustering *via* paired group UPGMA algorithm in the PAST software (Hammer *et al.*, 2001) to graphically represent the species complexity between days. The relative abundance data of the gut microbes at each taxonomic level on different days was compared with the control fish using the independent *T*-test/the Mann–Whitney U test based on the normality of the data. These analyses were performed using SPSS (version 16), where *P*-value of <0.05 was set to represent the significant difference. Contributions of each taxonomy assigned bacterial OTU to the difference between gut bacterial communities of the treated fish group and the control group at different days of treatment were analyzed with the Similarity Percentage (SIMPER) analysis using the PAST software. The OTU abundance data accounting for >0.1% contribution to the dissimilarity in SIMPER analysis was used to generate the heatmap. The results of the PICRUSt2 analysis were also applied in the SIMPER analysis to determine the KEGG genes and pathways, which contributed most to the discrimination of samples in each day compared to the control group. The abundance data of KEGG genes and pathways contributing to >0.1% dissimilarity in SIMPER analysis on each day was used to generate the heat map and compared with the control fish using the independent *T*-test/the Mann–Whitney U test based on the normality of the data.

## Results

### Survival Rates

The fish in both the control and treatment groups showed 100% survival throughout the experimental period. Further, no clinical abnormalities were observed throughout the study period.

### Enumeration of Total Cultivable Bacteria and Presumptive Vibrios in the Gut

**FIGURE 1:**
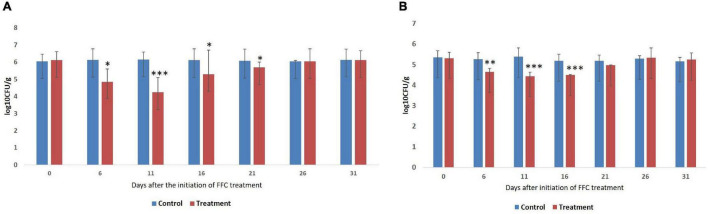
Enumeration of gut bacteria during different days of therapeutic exposure. **(A)** Enumeration of total viable counts of gut bacteria. **(B)** Enumeration of presumptive vibrio counts. Average log_10_ CFU per gram of gut tissue ± SE is shown in Y-axis. *P*-values less than 0.05, 0.01, and 0.001 are summarized with one, two, and three asterisks respectively, to represent the significant difference levels compared to the control animals. CFU, colony-forming units; FFC, florfenicol.

The results of the enumeration of gut microbes during different days of FFC exposure are presented as log colony forming units (log_10_ CFU ± SE) per gram of gut tissue (Figure 1). The gut of the control fish showed 6.1 ± 0.05 and 5.38 ± 0.07 cultivable bacteria in ZMA and TCBS, respectively. The Tukey *post hoc* test revealed that there was no significant difference in the CFU value between different days of the experiment within the control fish. However, there was a significant difference (*P* < 0.05) in the CFU values of the FFC treated fish between different days. The least bacterial count in both the media was observed in FFC treated fish on zero-day post-withdrawal (11th day after the initiation of the treatment) (*P* < 0.001). Further, the total *via*ble bacterial counts on the fifth-day post-initiation and fifth and 10th-day post-withdrawal of FFC treatment were significantly lower than the control fish (*P* < 0.05) (Figure 1A). The presumptive vibrio counts on the fifth-day post-initiation and fifth-day post-withdrawal of FFC treatment were significantly lower than the control fish (*P* < 0.05) (Figure 1B). In other words, the total viable count and presumptive vibrio counts were similar to the control group (*P* > 0.05) by 10 and 5 days, respectively, post-withdrawal of FFC treatment.

### Enumeration of Antimicrobial-Resistant Gut Bacteria

**FIGURE 2:**
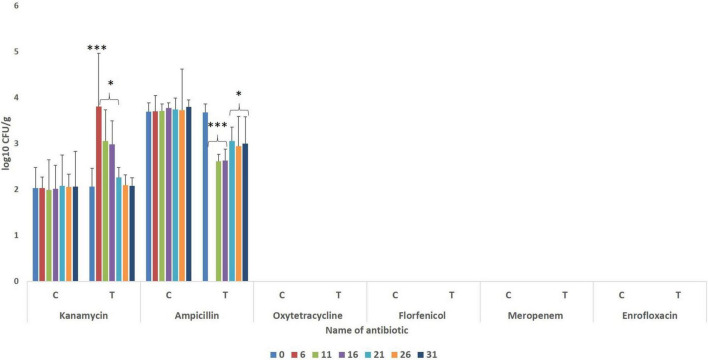
Enumeration of antibiotic-resistant bacteria during different days of FFC exposure. Average log_10_ CFU per gram of gut tissue ± SE is shown in Y-axis. *P*-values less than 0.05, 0.01, and 0.001 are summarized with one, two, and three asterisks, respectively, to represent the significant difference levels compared to the control animals. Different days after initiation of FFC treatment are shown in different color bars. CFU, colony-forming units; FFC, florfenicol; C, control fish; T, FFC treated fish.

The gut of the control fish revealed the presence of bacteria having resistance to ampicillin and kanamycin (Figure 2), with significantly higher numbers of ampicillin-resistant bacteria. During the FFC therapeutic course (fifth-day post-initiation), ampicillin-resistant microbes could not be detected in the treated group. Further, the numbers of ampicillin-resistant gut bacteria were significantly lower (*P* < 0.05) than the control fish after 20 days post-withdrawal of the treatment. There was a significant (*P* < 0.05) but transient increase in the numbers of kanamycin-resistant bacteria in the FFC treated fish than in the control group at five days post-initiation, and 5- and 10-days post-withdrawal of FFC treatment. More importantly, there was no growth in the FFC supplemented media in both control and the FFC treated fish even at zero-day post-withdrawal of treatment.

### Metagenomic Library Preparation and Sequencing

The parameters recorded during metagenomic library preparation and sequencing are shown in Supplementary Table 1. All the samples were qualified for library preparation and sequencing. A total of 9, 31, 971 reads of *16S rRNA* sequence were achieved following the quality filtering through DADA2. The numbers of merged reads varied from 1,209 to 10,105. The α-rarefaction curve displayed that a sequencing depth of ∼1,095 was sufficient to capture the maximum diversity, so that the features at this depth were used for the analysis. The metagenomic data sets of the present study are deposited as Sequence Read Archive (SRA) data (Accession numbers: SRR16990455–SRR16990471) under the Bio project Accession No. PRJNA780352 in the National Center for Biotechnology Information database.

### Microbial Diversity in the Gut of Snubnose Pompano Juveniles

**FIGURE 3:**
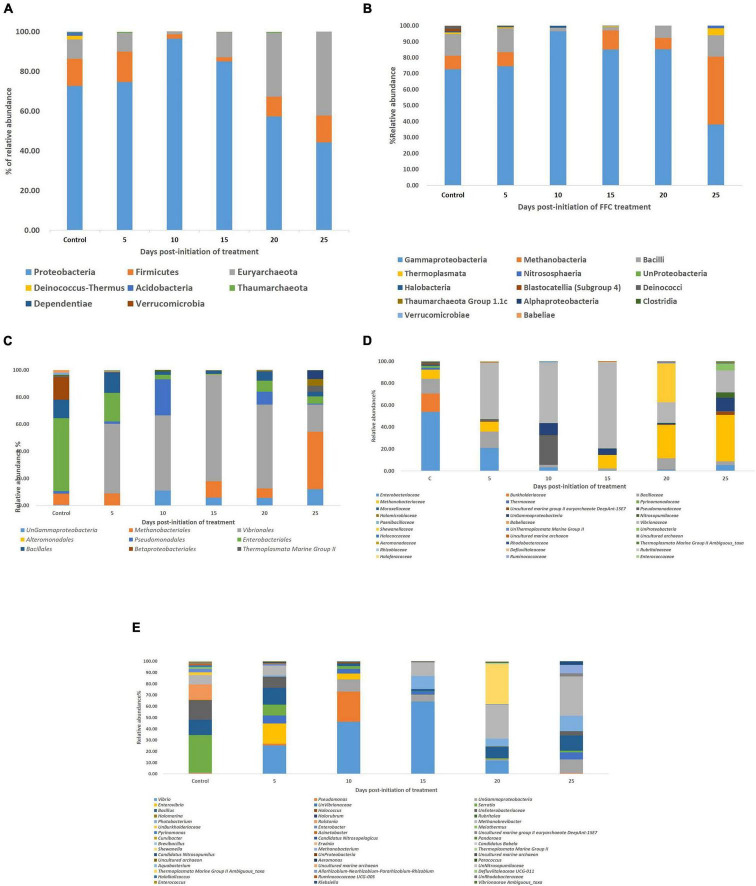
Impact of FFC therapeutic exposure on the gut microbiome taxonomy. **(A)** At phylum level; **(B)** at class level; **(C)** at order level; **(D)** at family level; **(E)** at genus level. FFC, florfenicol.

Taxonomic assignment of OTUs showed two identified distinct domains [Bacteria (91.66%), Archaea, (0.48%), and unassigned (7.94%)], 7 identified distinct phyla, 9 identified distinct classes, 12 identified distinct orders, 14 identified distinct families, and 20 identified distinct genera with ≥0.01% relative abundance. Proteobacteria (72.73%) occupied the maximum relative abundance among the identified phyla, followed by Firmicutes (13.64%), Euryarchaeota (9.83%), Deinococcus-Thermus (1.83%), Acidobacteria (1.54%), Thaumarchaeota (0.29%), and Dependentiae (0.14%) (Figure 3A). There were 11 distinct genera (≥0.01% relative abundance) within Proteobacteria in the order of *Serratia* sp. > Unassigned Enterobacteriaceae > *Ralstonia* sp. > *Enterobacter* sp. > Unassigned Burkholderiaceae > *Acinetobacter* sp. > *Pseudomonas* sp. > Unassigned γ-Proteobacteria > *Curvibacter* sp. > *Pandoraea* sp. > *Erwinia* sp. Even though the phylum Firmicutes was the second most dominant phylum after Proteobacteria, only two dominant genera could be identified, which were *Bacillus* sp > *Brevibacillus* sp. There were three identified genera in the phylum Euryarchaeota in the order of *Methanobrevibacter* sp. > Uncultured marine group II Euryarchaeote > *Halomarine* sp. Only one dominant genus, *viz. Meiothermus* sp., *Pyrinomanas* sp., and Candidatus *Nitrosopelagicus* sp., could be identified from the phylum Deinococcus-Thermus, Acidobacteria, and Thaumarchaeota, respectively.

### Dynamics of Microbial Diversity Measures Following Therapeutic Exposure of Florfenicol

**FIGURE 4:**
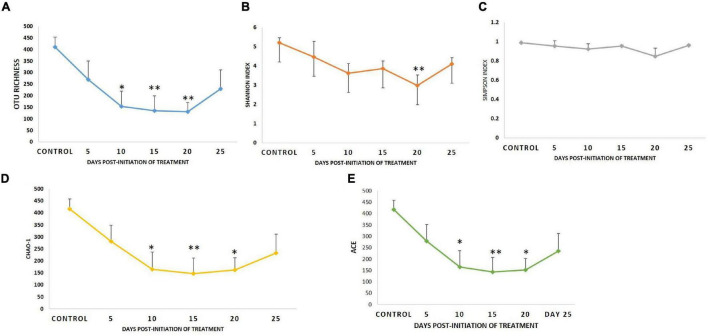
Dynamics of microbial diversity measures following therapeutic exposure of FFC. **(A)** Dynamics of OTU richness. **(B)** Dynamics of Shannon index. **(C)** Dynamics of Simpson index. **(D)** Dynamics of Chao-1. **(E)** Dynamics of abundance-based coverage estimator. *P*-values less than 0.05, 0.01, and 0.001 are summarized with one, two, and three asterisks, respectively, to represent the significant difference levels from the control animals.

**FIGURE 5:**
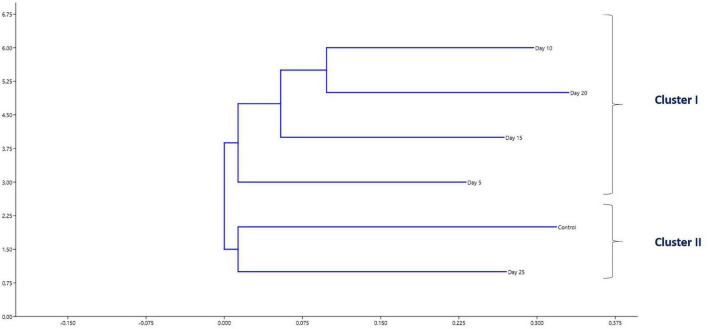
Hierarchical clustering based on the average Bray-Curtis similarity index.

Diversity analysis (α-diversity) of gut microbial communities revealed significant changes in all the diversity measures, except evenness and Simpson index, between different days (*P* < 0.05). All the gut microbial diversity measures were statistically similar in the FFC treated fish on the 5th day from the initiation of treatment and on the 15th day post-withdrawal to that of the control fish. The diversity measures of the control group, and FFC treated fish at 5 days from the initiation of treatment and on the 15th day post-withdrawal were found to be significantly higher than other groups (Figure 4). In other words, the fish belonging to zero, 5-, and 10-days post-withdrawal of FFC treatment had significantly lower diversity measures of the gut microbiome than the control group. Furthermore, within the treatment group, there was no significant difference (*P* > 0.05) in the gut microbial diversity measures on zero, 5-, and 10-days post-withdrawal of FFC treatment. The pattern in the dynamics of different diversity measures following the FFC treatment is shown in Figure 4. All the diversity measures were significantly decreased following FFC therapy and became similar to the control group at 15 days post-withdrawal. In other words, the differences in the gut microbial diversity measures became statistically indistinguishable in comparison to the control group on the 15th day post-withdrawal. PERMANOVA analysis based on the Bray–Curtis similarity index also demonstrated that the FFC treatment had a significant impact on the gut microbial communities of *T. blochii* (*P* = 0.05, F value = 1.312, Permutation number = 9,999, Total sum of squares = 6.04). The hierarchical clustering based on the average Bray-Curtis similarity index of each day showed that gut microbial communities of the fish on the 15th day post-withdrawal were clustered along with the control (Cluster II), while the others formed a distinct independent cluster (Cluster I) away from the first group (Figure 5).

### Impact of FFC Therapeutic Exposure on the Gut Microbiome Taxonomy

**FIGURE 6:**
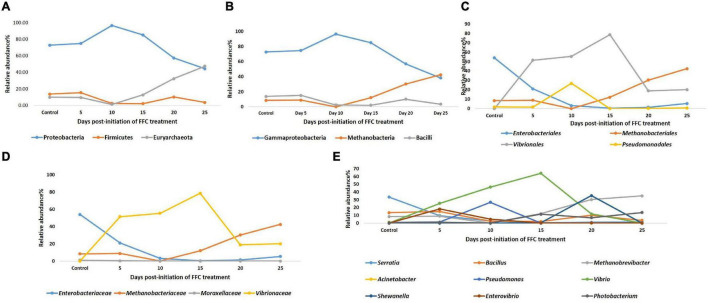
Dynamics of microbial taxon whose relative abundance was significantly altered by FFC treatment. **(A)** At phylum level; **(B)** at class level; **(C)** at order level; **(D)** at family level; **(E)** at genus level.

There were significant (*P* < 0.05) changes in the relative abundance of certain gut microbial taxon following FFC exposures compared to the control group (Figure 3). Further, there were considerable variations between the individual fish belonging to the same exposure period. Nevertheless, there was no significant difference between the treatment and control group in the relative abundance of microbes at the domain level on any day of FFC exposure. At the phylum level, the shift in microbiome communities was largely driven by OTUs assigned to the phylum Proteobacteria (Figure 3). The relative abundance of Proteobacteria was significantly increased at zero-day post-withdrawal, leading to the reduced representation of the other two phyla, namely Euryacrcheota and Firmicutes (Figure 6A). The changes in the phyla level became similar to the control group (*p* < 0.05) from 10 days post-withdrawal. At the class level, there was a significant (*P* < 0.05) transient increase and decrease in the relative abundance of γ-Proteobacteria and Methanobacteria, respectively, at zero-day post-withdrawal. The relative abundance of the class, Bacilli was also reduced at zero-day post-withdrawal (Figure 6B). The changes in different classes became similar to the control group at 15 days post-withdrawal. Among the major changes in the order level, the decrease in the relative abundance of Enterobacteriales and increase in Vibrionales remained even on the 15th day post-withdrawal (Figure 6C). At the family level, the relative abundance of Enterobacteriaceae and Moraxellaceae remained significantly at a lower level than the control fish at 15 days post-withdrawal (Figure 6D). At the genus level, there were several transient changes, however, the relative abundance of *Serratia* sp. and *Acinetobacter* sp. remained significantly at a lower level even at 15 days post-withdrawal (Figure 6E). It was striking to note that the relative abundance of *Vibrio* sp. showed an increasing trend from five days post-initiation of FFC treatment, reached the maximum level at five days post-withdrawal, and then showed a decreasing trend (Figure 6E). The details of the major changes in the gut microbial taxonomy are briefly represented in Table 1.

**TABLE 1:** Major changes from the control group in the gut microbial taxonomy following therapeutic exposure to florfenicol (FFC).

Microbial group	Days from the initial FFC exposure				
	5	10	15	20	25
**At domain level**	NC				
**At phyla level**					
Proteobacteria	NC	↑	NC	NC	NC
Firmicutes	NC	↓↓	↓↓	NC	NC
Euryacrcheota	NC	↓↓	NC	NC	NC
**At class level**					
γ-Proteobacteria	NC	↑	NC	NC	NC
Methanobacteria	NC	↓↓↓	NC	NC	NC
Bacilli	NC	↓↓	↓↓	NC	NC
**At order level**					
Methanobacteriales	NC	↓↓↓	↓	↓	NC
Enterobacteriales	NC	↓↓	↓↓↓	↓↓	↓↓
Vibrionales	↑↑↑	↑↑↑	↑↑↑	↑↑↑	↑↑
Pseudomonadales	NC	↑↑	NC	NC	NC
**At family level**					
Enterobacteriaceae	NC	↓↓	↓↓↓	↓↓↓	↓↓
Methanobacteriaceae	NC	↓↓↓	NC	NC	NC
Moraxellaceae	NC	↓↓↓	↓↓↓	↓↓↓	↓↓↓
Vibrionaceae	↑↑↑	↑↑↑	↑↑↑	↑↑↑	↑↑
**At genus level**					
*Serratia* sp.	NC	↑↑	↑↑↑	↑↑↑	↑↑↑
*Methanobrevibacter* sp.	NC	↓↓↓	NC	NC	NC
*Acinetobacter* sp.	NC	↓↓↓	↓↓↓	↓↓↓	↓↓↓
*Bacillus* sp.	NC	↓↓	↓↓	NC	NC
*Vibrio* sp.	↑↑↑	↑↑↑	↑↑↑	↑↑	NC
*Pseudomonas* sp.	NC	↑↑↑	NC	NC	NC
*Shewanella* sp.	NC	NC	NC	↑↑↑	NC
*Enterovibrio* sp.	↑↑	↑	NC	NC	NC
*Photobacterium* spp.	NC	NC	↑↑	↑↑	↑↑

*Red upward arrows indicate the increase in the relative abundance compared to control group, where one, two, and three arrows show the increase >5, 5–20, and >20 times, respectively higher than the control group. Green downward arrows indicate the decrease in the relative abundance from the control group, where one, two, and three arrows show the decrease >0.2, 0.2–0.05, and >0.05 times lower, respectively than the control group. NC, no significant change from the control; FFC, florfenicol.*

**FIGURE 7:**
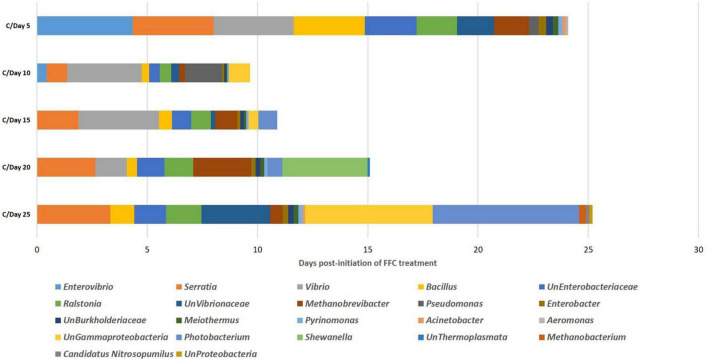
Similarity percentages analysis showing the microbial taxon contributing to the discrimination between different days of FFC exposure.

In the SIMPER analysis, there were 15, 14, 13, 13, and 16 differentiating identified genus-level taxa in the FFC-treated fish group compared to the control fish (>0.1% contribution to the dissimilarity) at 5, 10-, 15-, 20-, and 25-days post-initiation of the treatment (Figure 7). On the 5th day, the maximum dissimilarity in the treated fish group in comparison to the control group was caused by an increased abundance of *Enterovibrio* sp. and *Vibrio* sp. and decreased abundance of *Serratia* sp. At zero-day post-withdrawal, the maximum dissimilarity was caused by an increased abundance of *Vibrio* sp. and *Pseudomonas* sp. and decreased abundance of *Serratia* sp. At five-day post-withdrawal, the maximum dissimilarity of the FFC-treated fish compared to the control was caused by an increased abundance of *Vibrio* spp. and decreased abundance of *Serratia* spp. The maximum dissimilarity compared to the control fish at 10-day post-withdrawal was contributed by the increased abundance of *Shewanella* sp. and decreased abundance of *Serratia* sp. On the 15th day post-withdrawal, the increased abundance of *Photobacterium* sp. and decreased abundance of *Serratia* sp. contributed to the maximum dissimilarity in the FFC treated fish group compared to the control fish.

### Impact of Florfenicol Therapeutic Exposure on the Functional Metagenomics of Gut Microbiome

There were 62, 51, 56, 114, and two differentiating KEGG genes at 5, 10, 15, 20, and 25 days, respectively, of therapeutic exposure with >0.1% contribution. Of which, the abundances of 45 and 44 KEGG genes were significantly different in the FFC treated fish group compared to the control group, at zero and five days of the FFC withdrawal (Table 2). However, none of the genes were significantly different in the treated group in comparison to the control group at five days post-initiation of treatment, and 10 and 15 days of FFC withdrawal. In other words, the abundance of KEGG genes returned to the control group level by 10 days of FFC withdrawal. At zero-day post-withdrawal, the maximum significant dissimilarity compared to the control group was caused by the increased abundance of K03406 (methyl-accepting chemotaxis protein), K02030 (polar amino acid transport system substrate-binding protein), K00059 (3-oxoacyl-[acyl-carrier protein] reductase), K02014 (Ferrichrome outer membrane transporter), and K02015 (iron ABC transporter permease). Further, the significant increases in the abundances of two multidrug-resistant proteins, *viz.* K03327 and K03585 at zero-day post-withdrawal were noteworthy. On the fifth day post-withdrawal, the maximum significant dissimilarity in comparison to the control group was caused by the increased abundance of K03406 (methyl-accepting chemotaxis protein), K02004 (Efflux ABC transporter permease protein), K02003 (ABC transporter system ATP binding protein), and K02015 (iron ABC transporter permease). It was interesting to note that there were no significant changes in the abundance of the genes involved in multidrug/antibiotic resistance from the fifth-day post-withdrawal onward.

**TABLE 2:** Details of KEGG genes whose abundances were significantly different compared to the control fish.

Sl. No	Zero-day post-withdrawal			Five-day post-withdrawal		
	KEGG ID	Name of gene	% Contribution to dissimilarity	KEGG ID	Name of gene	% Contribution to dissimilarity
1.	K03406	*mcp* (methyl-accepting chemotaxis protein gene)	0.4268	K03406	*mcp*	0.3137
2.	K02030	ABC-PAS (polar amino acid transport system substrate-binding protein gene)	0.2646	K02004	ABC-CDP (putative ABC transport system permease protein)	0.2243
3.	K07090	K07090 (uncharacterized protein)	0.248	K07090	K07090	0.2098
4.	K00059	*fabG* {(3-oxoacyl- [acyl-carrier protein] reductase}	0.2186	K02003	ABC-CDA (putative ABC transport system ATP-binding protein)	0.2056
5.	K02014	TC.FEV.OM (iron complex outer membrane receptor protein)	0.2168	K02015	ABC.FEV.P (iron complex transport system permease protein)	0.1992
6.	K02015	ABC.FEV.P	0.1995	K01992	ABC-2. P (ABC-2 type transport system permease protein)	0.1788
7.	K03088	*rpoE* (RNA polymerase sigma-70 factor, ECF subfamily)	0.1912	K02342	*dnaQ* (DNA polymerase III subunit epsilon)	0.1756
8.	K03704	*cspA* [cold shock protein (β-ribbon, CspA family)]	0.1905	K03561	*exbB* (biopolymer transport protein ExbB)	0.1641
9.	K02004	ABC.CD.P	0.1839	K06147	ABCB-BAC (ATP-binding cassette, subfamily B, bacterial)	0.1605
10.	K02003	ABC.CDA	0.1807	K06076	*fadL* (long-chain fatty acid transport protein)	0.1598
11.	K01992	ABC-2. P	0.1641	K03704	*cspA*	0.1502
12.	K02342	*dnaQ* (DNA polymerase III subunit epsilon)	0.161	K02016	ABC.FEV.S (iron complex transport system substrate-binding protein)	0.1487
13.	K02013	ABC.FEV.A (iron complex transport system ATP-binding protein)	0.1585	K00059	*fabG*	0.1484
14.	K00626	E2.3.1.9, *atoB* (acetyl-CoA C-acetyltransferase)	0.1567	K02032	ABC.PE.A1 (peptide/nickel transport system ATP-binding protein)	0.1448
15.	K02016	ABC.FEV.S	0.1559	K02013	ABC.FEV.A	0.1436
16.	K03415	*cheV* (two-component system, chemotaxis family, chemotaxis protein CheV)	0.1409	K08303	Putative protease	0.1432
17.	K02002	*proX* (glycine betaine/proline transport system substrate-binding protein)	0.1394	K02030	ABC.PA.S	0.1407
18.	K03561	*exbB* (biopolymer transport protein ExbB)	0.1373	K07114	*yfbK* (Ca-activated chloride channel homolog)	0.1393
19.	K02029	ABC.PA.P (polar amino acid transport system permease protein)	0.1321	K03088	*rpoE* (RNA polymerase sigma-70 factor, ECF subfamily)	0.1367
20.	K03310	TC. AGCS (alanine or glycine: cation symporter, AGCS family)	0.1287	K03310	TC. AGCS	0.1364
21.	K07025	Putative hydrolase of HAD superfamily	0.1284	K02035	ABC.PE.S (Peptide/nickel transport system substrate-binding protein)	0.1328
22.	K03424	*tatD* (TatD-DNase family protein)	0.128	K02040	*PstS* (Phosphate transport system substrate-binding protein)	0.1291
23.	K06076	*fadL* (long-chain fatty acid transport protein)	0.1255	K07025	Putative hydrolase of HAD superfamily	0.1275
24.	K06147	ABCB-BAC	0.1243	K03424	*tatD*	0.1268
25.	K07107	*ybgC* (acyl-CoA thioester hydrolase)	0.1235	K03566	*gcvA* (LysR family transcriptional regulator, glycine cleavage system transcriptional activator)	0.1235
26.	K02040	*pstS* (Phosphate transport system substrate-binding protein)	0.1227	K03415	*cheV* (Two-component system, chemotaxis family, chemotaxis protein CheV)	0.1141
27.	K07114	*yfbK*	0.1209	K02495	*hemN*, *hemZ* (oxygen-independent coproporphyrinogen III oxidase)	0.1113
28.	K01626	E 2.5.1.54, aroF, aroG, aroH; 3-deoxy-7-phosphoheptulonate synthase	0.1156	K01626	E2.5.1.54, aroF, aroG, aroH; 3-deoxy-7-phosphoheptulonate synthase	0.1076
29.	K03585	*acrA, mexA, adeI, smeD, mtrC, cmeA*; (Membrane fusion protein, multidrug efflux system)	0.1153	K01990	ABC-2. A; ABC-2 type transport system ATP-binding protein	0.1075
30.	K02035	ABC.PE.S	0.1144	K06177	*rluA* (tRNA pseudouridine32 synthase/23S rRNA pseudouridine746 synthase)	0.1015
31.	K00134	GAPDH, *gapA* (glyceraldehyde 3-phosphate dehydrogenase)	0.1133	–	–	–
32.	K02495	*hemN, hemZ*	0.1131	–	–	–
33.	K03566	*gcvA*	0.112	–	–	–
34.	K03408	*cheW* (Purine-binding chemotaxis protein CheW)	0.1119	–	–	–
35.	K02032	ABC.PE.A1	0.1106	–	–	–
36.	K03719	*lrp* (Lrp/AsnC family transcriptional regulator, leucine-responsive regulatory protein)	0.1101	–	–	–
37.	K03924	*moxR* (MoxR-like ATPase)	0.1084	–	–	–
38.	K01990	ABC-2. A	0.1083	–	–	–
39.	K06177	*rluA*	0.1083	–	–	–
40.	K03496	*parA, soj* (chromosome partitioning protein)	0.1075	–	–	–
41.	K00020	*mmsB* (HIBADH; 3-hydroxyisobutyrate dehydrogenase)	0.104	–	–	–
42.	K02415	*fliL* (flagellar FliL protein)	0.103	–	–	–
43.	K03321	TC. SULP (sulfate permease, SulP family)	0.1025	–	–	–
44.	K03327	TC. MATE, SLC47A, norM, mdtK, dinF; multidrug resistance protein, MATE family	0.1022	–	–	–
45.	K08303	Putative protease	0.1005	–	–	–

There were 235, 205, 207, 206, and 174 differentiating KEGG pathways at 5, 10, 15, 20, and 25 days of therapeutic exposure with >0.1% contribution, respectively. Of which, the abundances of 97 and 173 KEGG pathways were significantly different in comparison to the control group, at zero and five days of the FFC withdrawal (Table 2). However, the abundances of KEGG pathways at five days post-initiation of treatment, and 10 and 15 days of FFC post-withdrawal were similar to that of the control group (*P* > 0.05). In other words, the abundance of KEGG pathways also became similar to the control group by 10 days post-withdrawal of FFC treatment. At zero-day post-withdrawal, the maximum significant dissimilarity compared to the control was caused by the increased abundance of PWY3781 (aerobic respiration/cytochrome c), PWY7663 (gondoate biosynthesis: anaerobic), FAS YN-ELONG-PWY (fatty acid biosynthesis), FAO PWY (fatty acid oxidation), and PWY 7664 (oleate biosynthesis IV anaerobic). On the fifth day post-withdrawal, the maximum significant dissimilarity compared to the control was caused by the increased abundance of PWY7663 (gondoate biosynthesis: anaerobic), FAO PWY (fatty acid oxidation), and PWY 5989 (stearate biosynthesis II).

## Discussion

Antibiotics play a significant role in treating and controlling bacterial diseases, which are a major impediment to the economic sustainability of aquaculture (Pridgeon, 2012). The antibiotic treatment options are critically crucial for farmed tropical marine fishes like snubnose pompano (*T. blochii*), where alternative prophylactic strategies such as vaccines are completely absent. Contrariwise, cutting-edge research in terrestrial animals has amply demonstrated the adverse effect of antibiotic treatment in disrupting the healthy gut microbiome of the host (Holman et al., 2019). As the gut microbiome serves several vital biological and physiological functions for the host, understanding the intended and unintended consequences of antibiotic treatment on the gut microbiome is vital to support the overall health and welfare of the farmed animals (Payne et al., 2021). The antibiotic treatment can also cause the emergence of antimicrobial resistance (AMR) in gut microbes, raising additional concerns about consumer and environmental safety (Cabello et al., 2013). The effect of antibiotic treatment on the gut microbiome of marine fish has not been studied, so far, despite its increasing relevance from both agricultural and ecological perspectives. In this context, the present study was envisaged to ascertain the impact of recommended therapeutic dose of FFC, one of the FDA-recommended antimicrobial compounds for aquaculture use, on the gut microbiome of *T. blochii*. The study forms the first report on the unintentional consequences of an approved antibiotic treatment on the gut microbiome of a marine fish species.

Preliminary evaluation using culture-dependent methods showed that the FFC treatment significantly (*P* < 0.05) reduced the gut bacterial density even from 5 days post-initiation of the treatment. This was an expected result, as feeding antibiotics were shown to inhibit the normal intestinal microbiota of fish (Gaskins et al., 2002). There was a two log-reduction in the total viable bacterial count compared to the control fish gut at zero-day post-withdrawal. A similar observation was reported in zebrafish, where colistin and vancomycin treatment reduced the total viable count by 3–5 logs and 2 logs, respectively (Brugman et al., 2009). More importantly, in the present study, the total viable bacteria and presumptive vibrios became similar to the control group by the 10th and 5th-day post-FFC withdrawal, respectively. These results suggested that the therapeutic exposure to FFC could induce significant short-term changes to the cultivable gut microbiome, followed by its restoration at the 10th-day post-withdrawal. The findings were supported by Kim et al. (2019), who observed the restoration of the cultivable bacterial load in olive flounder (*Paralichthys olivaceus*) on the 10th day after treatment with the therapeutic dose of oxytetracycline and amoxicillin.

The evaluation using 16SrRNA amplicon-based metagenomics approach was followed for a detailed understanding of the treatment consequences on the gut microbiome. The results of α-diversity metrics showed that the FFC-treated fish had significantly lower diversity and richness measures of gut microbiome than the control group at 0-, 5-, and 10-days post-withdrawal of FFC-medicated feed. Earlier studies on antibiotic-induced perturbations in the commensal microbes of the freshwater fish gut and aquatic environments have also revealed the reduction in the gut microbial diversity measures following either FFC or oxytetracycline treatment (He et al., 2011; Navarrete et al., 2008; Kim et al., 2019; Wang et al., 2019; Zeng et al., 2019). Using hierarchical clustering, the visual representation of β−diversity measures reflected a clear separation between FFC-fed and control samples. More strikingly, both alpha and beta diversity measures showed a similarity between the gut microbiome of the FFC-fed and control fish at 15 days post-withdrawal, illustrating the restoration of the gut microbiome at 15 days of post-withdrawal of medicated feed in the test groups. While the difference in the gut bacterial density between FFC-treated and control fish was evident from the fifth-day post-initiation of treatment in culture-dependent methods, and no dissimilarity was observed in culture-independent methodologies based on α- and β-diversity measures. The ineptitude of DNA-based culture-independent methodologies to differentiate between the dead and viable bacteria in the gut can be attributed to the above observation. In support of our findings, Reikvam et al. (2011) reported that DNA-based methodologies could not detect the reduction in microbial load as early as those observed by culture-dependent techniques.

To shed more light on the FFC treatment-induced gut microbial dysbiosis, the changes in the relative abundance of different OTUs were analyzed. The results revealed certain significant changes in the relative abundance of different gut microbial taxons. At the phylum level, there was a significant increase in the relative abundance of Proteobacteria in the FFC-treated group, along with a significant reduction in the relative abundance of Euryacrcheota and Firmicutes. Increased abundance of Proteobacteria and decreased abundance of Firmicutes in the fish gut and aquaculture environment of FFC-treated freshwater fish have also been reported in earlier studies (Abdelhamed et al., 2019; Zeng et al., 2019). The studies on transgenic fast-growing common carp (*Cyprinus carpio* L.) showed that the increased relative abundance of Firmicutes could confer a fast growth to the fish (Li et al., 2013). Even though the effects of dietary FFC on the weight gain of fish were not recorded in the present study, Gaikowski et al. (2013) demonstrated a significant reduction in the bodyweight of FFC-treated tilapia (*Oreochromis* sp.). Taken together, the decreased relative abundance of Firmicutes observed in the present study might be a novel explanation for the reductions in body weight of FFC-treated tilapia observed by Gaikowski et al. (2013) and warrant future investigation to confirm the hypothesis. Another interesting observation in the microbial shift during downstream analysis was the significant reduction in the relative abundance of Enterobacteriaceae. As many members of Enterobacteriaceae have been reported to benefit from the host metabolic activity and nutrient utilization (Wu et al., 2012), the observed decrease can be another possible novel explanation for the reductions in bodyweight of the FFC-treated fish reported in previous studies.

At the class level, there was a significant transient increase in the relative abundance of γ-Proteobacteria and decreased abundance of Bacilli at zero-day post-withdrawal but became similar to the control group from fifth-day post-withdrawal in the case of γ-Proteobacteria and 10th-day post-withdrawal in the case of Bacilli. The γ-Proteobacteria comprise most opportunistic fish pathogens and tend to increase after exposure to different stressors in fish (Austin and Austin, 2007; Boutin et al., 2013; Webster et al., 2021). The results reinforced the earlier hypothesis of Webster et al. (2021) that an increased abundance of γ-Proteobacteria in the fish gut can be a common signature of certain stress exposure. On further downstream analysis, the reduced abundance of Firmicutes was linked to a reduction in the abundance of *Bacillus* sp. Whereas, the increased abundance of γ-Proteobacteria was related to an increase in the abundance of *Vibrio* sp., *Enterovibrio* sp., *Photobacterium* sp., *Pseudomonas* sp., and *Shewanella* sp. As these genera represent significant opportunistic marine fish pathogens (Mohamad et al., 2019), the transient rise observed in their relative abundance for a period of five-day post-withdrawal of the FFC suggested the possible increase in the susceptibility of the treated fish to different opportunistic diseases caused by them. The results warrant applying health management measures, like probiotics during the FFC withdrawal period, to reverse the observed negative effect as done by Schmidt et al. (2017), following streptomycin treatment in a freshwater fish species, *Poecilia sphenops*. In this context, it is noteworthy that the microbes belonging to Vibrionaceae can grow in TCBS agar (Bolinches et al., 1988), and the observed reduction of the bacterial count on the TCBS agar through culture-dependent mehods in the present study (from fifth-day post-initiation to the fifth-day post-withdrawal of FFC treatment), showed that the increase observed in Vibrionaceae was only a relative increase. In short, the results showed that FFC treatment caused a reduction in all the gut microbial counts but there was a clear shift in the gut microbiome toward well-known putative pathogens. In support of our findings, the shifts in the gut microbiome toward well-known putative pathogens following different antibiotic treatments were reported in the earlier studies on freshwater fish (Zhou et al., 2018; Sáenz et al., 2019; Payne et al., 2021). Simultaneously, the observed reduction in the bacterial count on TCBS agar showed that FFC has good antagonistic activity against the major marine fish pathogens (belonging to Vibrionacetheae family).

Further, different OTUs affiliated with *Serratia* sp., *Methanobrevibacter* sp., *Acinetobacter* sp., and *Bacillus* sp. showed reduction for a brief period in the FFC-treated fish gut. The possible role of *Methanobrevibacter* sp. in fiber digestibility in swine (Niu et al., 2015) and the potential probiotic activity of *Acinetobacter* sp. and *Bacillus* sp. in fish (Pandey et al., 2011; Tarnecki et al., 2019; Kuebutornye et al., 2020) have been reported. The observed reversal of their abundance within 20 days post-FFC treatment suggested the transient nature of dysbiosis following the antibiotic treatment. The gut microbiome research in humans has demonstrated that the use of certain probiotic strains could reduce antibiotic-associated diseases (Szajewska and Kołodziej, 2015). Accordingly, the reduced specific microbial signatures in the present study must be explored in the future for developing microbial management measures/probiotic dietary supplements to improve fish health during the antibiotic withdrawal period.

An important observation from the present study was the complete restoration of the gut microbiome in terms of cultivable bacterial load, diversity measures of metagenomics, functional metagenomic profiles, and taxonomic composition up to class level within 10–15 days of FFC withdrawal. However, at the lower taxonomic level (order, family, and genus), microbial composition remained changed at 15 days post-withdrawal. More specifically, the changes observed in the relative abundance of Vibrionaceae (*Vibrio* sp. and *Photobacterium* sp.), Enterobacteriaceae (*Serratia* sp.), and Moraxellaceae (*Acinetobacter* sp.) remained at 15 days of post-withdrawal. Even though restoration of cultivable gut microbial count by 10 days post-withdrawal of antibiotic treatment was reported by Kim et al. (2019), no previous study has evaluated the restoration dynamics of the gut microbial composition following antibiotic treatment in fish. In similar studies on human and mice gut microbiome, the bacterial load and diversity measures reversed over time after antibiotic therapy (Dethlefsen et al., 2008; Looft and Allen, 2012; Michelle et al., 2019), which supports findings. The implication of the observed short and long-term changes in the gut microbiome profiles on the long-term host’s health and metabolism are also warranted in the future and may enable the development of new therapeutic/prophylactic strategies. Further, the impact of FFC on the gills and skin microbiome, intestinal integrity, and health and immunity indices of fish, as well as the forces that shaped the restoration of gut microbiome composition during the post-withdrawal period, are interesting topics for future investigation.

To have more information on gut microbial dysbiosis in terms of AMR, the enumeration of gut bacteria in different antibiotic-embedded plates was done. Interestingly, the FFC treatment could significantly reduce the numbers of ampicillin-resistant microbes in the gut of treated fish, which persisted even after 20 days post-withdrawal of the treatment. The results suggested that FFC treatment can be used against infections caused by ampicillin-resistant microbes. In accordance with our results, Maaland et al. (2015) pointed out that FFC and chloramphenicol can be used to treat infections caused by extended-spectrum β-lactamase producing bacteria. More strikingly, it was noted that the therapeutic exposure to FFC did not induce the emergence of FFC resistant bacteria in the gut of the treated fish. The observation further confirms the concept that the excessive/indiscriminate use of antibiotics, not the therapeutic dose, increases the likelihood of resistance acquisition by the gut microbiota (Francino, 2016). However, there was a significant transient increase in the numbers of kanamycin-resistant bacteria in the FFC treated fish up to 10 days post-withdrawal of the treatment, suggesting that an indirect transient selection of kanamycin-resistant bacteria occurred as a secondary effect of FFC treatment. Even though kanamycin treatment was shown to promote short-term resistance against streptomycin, tetracycline, and ampicillin (Chen et al., 2009), the interaction between FFC and kanamycin has not been studied to date. To find a probable reason for the observation, the results of PICRUSt (Langille et al., 2013) were thoroughly scrutinized. The results revealed a set of gut microbial KEGG genes and pathways related to different transporter systems, cell metabolism, biosynthesis, cell motility, SOS response, and extracellular structure, which were significantly altered by the FFC treatment. Among these, the significant but transient increase in the abundances of two multidrug-resistant proteins, *viz.* K03327 (a multidrug resistance protein belonging to the MATE family) and K03585 (membrane fusion protein belonging to multidrug efflux system) at zero-day post-withdrawal were noteworthy. The results suggest that FFC treatment could transiently select multidrug resistance due to the increase in multiple efflux pump systems. In consonance with our findings, Looft et al. (2012) found that several resistance genes unrelated to the exposed antibiotic were enriched in the swine gut microbiome following antibiotic exposure. More importantly, the changes in the abundance of all the KEGG genes and pathways of gut microbes were only transient and became similar to the control group by the 10th-day post-withdrawal. Altogether, the results suggested that the therapeutic dose of FFC did not cause an irreversible increase in the antibiotic resistance profiles of the gut microbiota. Furthermore, it did not promote the FFC resistant microbes in the gut of the treated fish. However, the transient increase in the numbers of kanamycin-resistant microbes and the abundance of multidrug resistance encoding genes observed in the present study warrant certain *bona fide* strategies for processing aquaculture effluents during the first 10 days post-withdrawal period of antibiotic therapy for avoiding the probable emergence and dispersal of antimicrobial-resistant bacteria in the environments. Further, the results warrant future investigations on the changes in the frequency of different AMR genes and applications of shotgun metagenomic methods to profile whole microbial genomes to have a thorough understanding of the impacts of FFC use in aquaculture on the dispersal of AMR phenomenon in the environment.

## Conclusion

The present study forms the first report on the modulation and restoration dynamics of gut microbiota following the oral therapeutic dosing of FFC in a high-value marine aquaculture candidate fish species. The results showed a complete restoration of the gut microbiome within 10-15 days of FFC withdrawal except for a few changes in the lower microbial composition. The results highlighted the need for implementing better stress-management measures during the initial days of the withdrawal period. The study also pointed out the possible microbial signatures of stress in the fish and possible probiotic microbes that can be explored to design fish health improvisation strategies during the withdrawal period. The results also suggested that the therapeutic exposure to FFC did not cause an irreversible increase in the antibiotic resistance profiles of the gut microbiota. Further, it did not promote the FFC resistant microbes in the gut of the treated fish. In brief, the paper generates interesting insights on the implications of FFC treatment in a marine fish species, targeting its applications to formulate the identified risk minimization strategies during sustainable aquaculture practices. Further, the results emphasize the need to implement better infectious disease management measures in aquaculture facilities and recommend restriction of the antimicrobial treatment for the inevitable situations with the specified therapeutic dose and duration only.

## Data Availability Statement

The data presented in the study are deposited in the NCBI repository, as Sequence Read Archive (SRA) data (accession numbers: SRR16990455–SRR16990471) under the Bio project accession no. PRJNA780352.

## Ethics Statement

The animal study was reviewed and approved by the Institute Animal Ethics Committee of ICAR-CMFRI, Kochi, India (Grant No: CIBA/AINP-FH/2020-21).

## Author Contributions

KS conceptualized the presented idea, supervised the project, and acquired financial support for the project leading to this publication. TS and KS supervised the findings, analyzed the results, and wrote the manuscript. GS performed the bioinformatics analysis of the data. SG and VP conducted the experiments, sampling, and sample processing. PA, PS, and MA provided technical support to carry out the experiments. PP and AG provided critical feedback while drafting the manuscript. All authors contributed to the article and approved the submitted version.

## Conflict of Interest

The authors declare that the research was conducted in the absence of any commercial or financial relationships that could be construed as a potential conflict of interest.

## Publisher’s Note

All claims expressed in this article are solely those of the authors and do not necessarily represent those of their affiliated organizations, or those of the publisher, the editors and the reviewers. Any product that may be evaluated in this article, or claim that may be made by its manufacturer, is not guaranteed or endorsed by the publisher.
