# Supplementary material for: Dysbiosis and Restoration Dynamics of the Gut Microbiome Following Therapeutic Exposure to Florfenicol in Snubnose Pompano (Trachinotus blochii) to Aid in Sustainable Aquaculture Production Strategies
Source: Front Microbiol. 2022 May 30;13:881275. doi: 10.3389/fmicb.2022.881275 (PMC9189426; doi:10.3389/fmicb.2022.881275)
Supplement: Supplementary file 1 [file Table_1.DOCX]

Supplementary Table 1: Parameters recorded during metagenomic library preparation and sequencing

| Sl. No | Criteria | Control fish | Days post initiation of treatment | | | | |
| --- | --- | --- | --- | --- | --- | --- | --- |
|  |  |  | 5 | 10 | 15 | 20 | 25 |
| 1 | DNA concentration (ng/µL) | 23.91 ± 27.00 | 12.24 ± 18.03 | 8.88 ± 12.96 | 0.52 ± 0.11 | 0.21 ± 0.04 | 7.69 ± 6.56 |
| 2 | *16SrRNA* PCR product (ng) | 38.85 ± 5.30 | 51.00 ± 8.40 | 102.30 ± 53.87 | 130.80 ± 68.08 | 67.70 ± 20.36 | 101.90 ± 89.38 |
| 3 | Phred score | 35.79 ± 0.06 | 35.83 ± 0.22 | 36.08 ± 0.06 | 35.98 ± 0.05 | 35.92 ± 0.14 | 35.92 ± 0.37 |
| 4 | Number of bases having Phred score >30 | 20.63 ± 0.25 | 16.16 ± 3.67 | 13.79 ± 2.92 | 13.94 ± 3.69 | 16.55 ± 3.54 | 16.08 ± 3.34 |
| 5 | Number of reads having Phred score >30 | 82489.00 ± 981.46 | 64632 ± 14673.15 | 55182.67 ± 11673.04 | 55771.67 ± 14749.47 | 66205 ± 14179.05 | 64300.67 ± 13372.47 |
| 6 | GC (%) | 53.25 ± 0.31 | 53.31 ± 0.13 | 52.88 ± 0.51 | 53.09 ± 0.05 | 53.38 ± 0.23 | 51.70 ± 1.75 |
